# Remarks on the Erysipelatous Inflammation Produced by the Juice of Rhus Toxicodendron

**Published:** 1826-02

**Authors:** John Frost

**Affiliations:** Member of the Royal Institution; Director of the Medical Botanical Society, &c.


ART. VI.—*Remarks on the Erysipelatous Inflammation produced by the Juice of Rhus Toxicodendron.* By JOHN FROST, Esq. F.L.S. Member of the Royal Institution; Director of the Medical Botanical Society, &c.

IT so rarely happens that cases of erysipelatous inflammation, produced by the juice of rhus toxicodendron, occur, that I have considered it incumbent on me to lay the particulars of two which have come under my notice before the profession.

The leaves of rhus toxicodendron, although retained in the materia medica list, are seldom used. Dr. Anderson, of Hull, has used them, in the form of powder, in cases of paralysis, in very small doses.

Before proceeding to detail the particulars of the cases, it may be remarked, that very many species of rhus produce, by coming in contact with the skin, great pain and inflammation. Gardeners not unfrequently have their hands *poisoned*, as they term it, by the rhus vernix; and, in pruning it, they are cautious not to allow the juice to touch them.

In visiting the garden of a physician in one of our northern counties lately, I saw rhus toxicodendron growing abundantly against a wall. On my making him acquainted with the acrimonious nature of the plant, he immediately ordered it to be rooted up, to prevent children or any person from being injured by it.

But to return to the object of this communication. I beg to observe that the first case was that of a man, who was employed to gather the leaves of rhus toxicodendron for the purpose of pharmacy; in doing which, some of the juice of the plant came in contact with his face, which it had no sooner touched than he began to complain of great pain. His face became reddened to an immense degree, accompanied by erysipelatous swelling, which increased rapidly to such an extent as nearly to deprive him of sight. His hands escaped injury in consequence of his having put on thick gloves, which he did from knowing the great acrimony of the juice of the leaf-stalks and twigs. Being in the garden at the time of the circumstance, I was called to him, and found him labouring under the effects I have just described. A brisk cathartic, consisting of calomel and jalap, was given him directly; and a lotion, consisting of Zinci acetatis, ℈ij.; Spiritus tenuioris, f.℥jss.; Tincturæ opii, f.ʒij.; Aquæ distillatæ, f.℥x. M. was frequently applied. Besides which, he had a draught, composed of Liquoris ammoniæ acetatis, f.ʒj.; Liquoris antimonii tartarizati, gtt. x.; Misturæ camphoræ, et Aquæ menthæ viridis, āā f.ʒvj. M. every six hours. This plan of treatment was adopted for the space of a week, with an occasional dose of opium at bed-time, with great advantage; but the swelling did not entirely subside till nearly a fortnight.

Since this case occurred, I had the particulars of another related to me by a medical man, who was unfortunately the subject of it, which are as follow: viz.—Being present at the gathering of the leaves of the plant, in the month of June, he took up some to examine their botanical characters, having taken the precaution of putting on his gloves previously to so doing. After a few minutes had elapsed, the weather being extremely warm, he took off his hat, and wiped the perspiration from his forehead with one of his gloves, which had imbibed a portion of the juice of the plant; not recollecting at the moment that he had but just been handling the foliage of rhus toxicodendron. Exactly the same symptoms ensued as in the former case, and were relieved by a similar plan of treatment.

These cases will prove that it is the *juice* of the plant which, when in contact with the skin, produces the effects above described; although a writer on the Materia Medica of the present day states, that "the stems (of rhus toxicodendron), if cut or broken, exude a milky juice, which was supposed to inflame the skin wherever it touched; but this is not the case, although the plant exudes a deleterious vapour," which it is said to do during the night season. How far this latter observation holds good with regard to the gathered foliage, may be estimated from the fact of a man being engaged for a whole day in spreading about and drying nearly thirty pounds of the fresh-collected plant, and not experiencing the slightest inconvenience.

The truth of the cases here related does not rest on my testimony only, as the parties are still living, and there are several scientific individuals who were witnesses of the effects.

